# Nigerian men and modern contraceptives: who are the non-users and what are their perceptions about family planning?

**DOI:** 10.11604/pamj.2023.46.64.37248

**Published:** 2023-10-19

**Authors:** Joshua Odunayo Akinyemi, Mobolaji Modinat Salawu, Rotimi Felix Afolabi, Ayo Stephen Adebowale

**Affiliations:** 1 Department of Epidemiology and Medical Statistics, Faculty of Public Health, College of Medicine, University of Ibadan, Ibadan, Nigeria,; 2 Demography and Population Studies Programme, Schools of Public Health and Social Sciences, University of the Witwatersrand, Johannesburg, South Africa

**Keywords:** Men, contraceptive use, perception, family planning, Nigeria

## Abstract

**Introduction:**

the decision of men is pertinent to contraceptive uptake in a patriarchal society like Nigeria. Earlier studies on contraception in Nigeria have focused majorly on women. In this paper, we identified factors influencing contraceptive use, and non-users’ perceptions about family planning among Nigerian men.

**Methods:**

using data from the 2018 Nigeria Demographic and Health Survey, this retrospective cross-sectional study focused on men aged 15-59 years. Three outcome variables were analysed: modern contraceptive use categorised as non-users or users; perception about contraception captured using two statements- “contraception is woman’s business”; “women who use contraception may become promiscuous”. Data were analyzed using multivariable logit model with robust standard errors (α= 0.05).

**Results:**

mean age of the men was 37.3 years (SD=10.2). Out of 9622 study participants, 71.0% do not use any modern contraceptive method; 19.9% believed that contraception is woman’s business while 38.1% believed that women who used contraceptives may become promiscuous. Significant predictors of non-use of contraceptives and perceptions about family planning were older age, low education, Islamic religion, exposure to family planning messages, desire for more children and residence in Northern part of Nigeria.

**Conclusion:**

large proportion of contraceptive non-users had negative perceptions. Educational intervention and advocacy among Nigerian men are essential to increase contraceptive uptake.

## Introduction

Contraceptive use is an important and effective population control measure. It impacts positively on nation development, economic growth, and maternal health by offering mothers enough time to recover from previous pregnancies, thus reducing maternal morbidity and mortality [1-3]. Contraception plays a critical role in birth spacing, thereby preventing short birth intervals which is a risk factor for childhood morbidity and mortality [4-6]. It provides a notable contribution to women´s autonomy and socio-economic empowerment through increased opportunity for education and, engagement in the workforce [7].

However, the Contraceptive Prevalence Rate (CPR) in sub-Sahara Africa (SSA) remained low as seventeen percent of women of the reproductive age group currently use contraceptives [8]. Fifteen percent of Nigerian women are currently using contraceptives, this is far below the country-set goal of 36% CPR [2,9]. The progress in increasing CPR in SSA since year 2000s has been slow due to some barriers among women such as lack of formal education, poverty, low empowerment status, and poor awareness about family planning [10,11].

Male involvement in contraception is another critical factor many family planning advocates have encouraged owing to its benefits and influence on contraceptive uptake [12]. This is very necessary among African men who are often heads of households and decision-makers on reproductive health matters such as the number of children the couple desires to have as well as the use and non-use of contraceptives [13-15].

Studies have shown that men´s knowledge as well as perception about contraceptive use influence their partners´ use of modern contraceptives. Most of these studies reported high awareness and knowledge of contraceptives among the males, but high unwillingness to allow spouses to use modern contraceptives [14,15]. Studies across Africa reported that negative perceptions about modern contraceptive use among men were the reasons for their lack of support for spouses to use [16-22].

While few previous studies in Nigeria have shown that certain beliefs among men are associated with contraceptive uptake, the beliefs/perceptions are rarely explored to understand their predisposing characteristics. There is also a dearth of information on male contraceptive non-users in Nigeria. It is important to know the peculiar characteristics of these non-users in order to provide evidence to further strengthen advocacy programs on contraception. This study was conducted to assess the perceptions of Nigerian men about modern contraceptives, determine the characteristics of non-users, and association between contraceptive perceptions and its non-use among Nigerian men aged 15-59 years.

## Methods

**Study design and setting:** this study was a retrospective analysis of nationally representative cross-sectional men´s data from the 2018 round of the Nigeria Demographic and Health Survey (NDHS). During the survey, households were selected using a stratified two-stage cluster sampling technique [3]. Men aged 15-59 years were interviewed in every second household selected. The total number of men interviewed was 13,311. In the men´s questionnaire for NDHS 2018, section 3 questions 301-307 collected data about contraception, and these include details such as knowledge of family planning methods, use of family planning, choice of method(s), sources of family planning, and attitude to family planning.

**Participants:** in this study, we excluded men who did not know any method of family planning (n=706) and those who never had sex (n=2983). Therefore, the final analytical sample was 9,657.

**Study variables:** we derived a dichotomous variable for modern contraceptive use categorized as non-users (1) or users (0). Data were also extracted for background characteristics and other variables such as the number of women fathered with, exposure to family planning messages. Perception about contraception was captured using two statements: (1) “contraception is a woman´s business and a man should not have to worry about it”; (2) “women who use contraception may become promiscuous”. The responses to these were either: “agree”, “disagree” or “don´t know”. These items were dichotomized as “agree” or disagree to capture negative and positive perceptions respectively. Don´t know was also classified as negative.

**Statistical analysis:** analysis was conducted in stages to address the research questions. First, cross-tabulation was done using frequencies and percentages to summarize the background characteristics of the study sample according to contraceptive use status. Next, we investigated the factors associated with negative perceptions among contraceptive non-users. Each perception was analyzed separately. Univariate and multivariable logistic regression models were fitted to estimate unadjusted and adjusted odds ratio (with 95% confidence interval). The final analyses were conducted to explore the independent relationship between contraceptive perceptions, background characteristics, and non-use of contraceptives among Nigerian men. For all analyses, we utilized sampling weight for descriptive results while robust standard errors were computed for multivariable models to account for the complex survey design of the NDHS.

**Ethical considerations:** informed consent was obtained from respondents during data collection and the survey protocol was approved by the National Health Research Ethics Committee in Nigeria [23]. Prior to the analysis, we obtained formal approval to download the NDHS 2018 from the online archive. The authors assert that all procedures contributing to this work comply with the ethical standards of the relevant national and institutional committees on human experimentation and with the Helsinki Declaration of 1975, as revised in 2008. The present study analysis utilized a secondary dataset, freely available for use in the public domain, which requires no ethics approvals. Meanwhile, the Demographic and Health Surveys Program, ICF Macro USA granted authorization to access the raw data set used for the present analysis.

## Results

**Background characteristics:** Table 1 presents the background characteristics of the study participants. About half of the respondents 4959 (51.3%) were aged 30-44 years and 2095 (21.7%) had no formal education while 4103 (42.5%) and 1913 (19.8%) attained secondary and tertiary education respectively. The majority of the men 5368 (55.6%) were engaged in agricultural/manual occupation. Results also showed that 4649 (48.1%) were urban dwellers. Across geopolitical regions, 2185 (22.6%) and 2154 (22.3%) were domiciled in the Northwest and Southwest respectively while the other four regions had between 1278 (13.2%) in the Southeast and 1354 (14.0%) in the Northeast. Half 4942 (51.2%) of the participants reported exposure to family planning messages. The majority (84.7%) were married with 6856 (71.0%) in monogamous relationships. Concerning fertility desire, about 6 out of 10 men want more children.

**Table 1:** background characteristics of Nigeria men who know about contraceptives, NDHS, 2018

Variables	All men: n(%)	Contraceptive users: n(%)	Non-users: n(%)	P-value
**Age group in years**				
15-29	2134 (22.1)	892 (41.4)	1242 (16.6)	<0.001
30-44	4959 (51.3)	931 (43.1)	4028 (53.7)	
45-59	2564 (26.6)	334 (15.5)	2230 (29.7)	
**Educational level**				
No formal education	2095(21.7)	103(4.8)	1992(26.6)	<0.001
Primary	1546(16.0)	225(10.4)	1321(17.6)	
Secondary	4103(42.5)	1191(55.2)	2912(38.8)	
Higher	1913(19.8)	638(29.6)	1275(17.0)	
**Occupation**				
Not working	377(3.9)	199(9.2)	178(2.4)	<0.001
Professional/services	2544(26.3)	732(33.9)	1812(24.2)	
Sales	1368(14.2)	272(12.6)	1096(14.6)	
Agriculture/manual	5368(55.6)	955(44.3)	4414(58.9)	
**Religion**				
Catholics	1212(12.6)	357(16.5)	855(11.4)	<0.001
Other Christians	3758(38.9)	1203(55.8)	2555(34.0)	
Islam	4605(47.7)	584(27.1)	4021(53.6)	
Others	82(0.9)	14(0.6)	68.3(0.9)	
**Wealth index**				
Poorest	1401(14.5)	87(4.0)	1314(17.5)	<0.001
Poorer	1612(16.7)	234(10.9)	1378(18.4)	
Middle	1930(20.0)	404(18.7)	1526(20.4)	
Richer	2186(22.6)	604(28.0)	1582(21.1)	
Richest	2528(26.2)	827(38.4)	1700(22.7)	
**Rural residence**	5009(51.9)	874(40.5)	4135(55.1)	<0.001
**Region**				
North Central	1333(13.8)	357(16.6)	976(13.0)	<0.001
North East	1354(14.0)	173(8.0)	1180(15.7)	
North West	2185(22.6)	196(9.1)	1989(26.5)	
South East	1278(13.2)	370(17.2)	908(12.1)	
South South	1353(14.0)	483(22.4)	870(11.6)	
South West	2154(22.3)	577(26.7)	1577(21.0)	
**Exposure to family planning messages**	4942(51.2)	1259(58.4)	3684(49.1)	<0.001
**Marital status**				
Never	1481(15.3)	989(45.8)	493(6.6)	<0.001
Monogamous	6856(71.0)	1025(47.5)	5830(77.7)	
Polygamous	1163(12.0	106(4.9)	1057(14.1)	
Former	157(1.6)	37(1.7)	120(1.6)	
**No of women fathered with**				<0.001
Never fathered	1847(19.1)	989(45.8)	858(11.4)	
One	6047(62.6)	976(45.2)	5071(67.6)	
Two or more	1763(18.3)	193(2.9)	1571(20.9)	
**Mobility (past 12 months)**				
Never away	4233(43.8)	847(39.3)	3386(45.1)	<0.001
Away < 1 month	3179(32.9)	748(34.7)	2431(32.4)	
Away > 1 month	2245(23.3)	562(26.1)	1683(22.4)	
**Fertility preference**				
Wants (more) children	5711(59.1)	680(31.5)	5031(67.1)	<0.001
Undecided	266(2.8)	45(2.1)	221(2.9)	
No more	2041(21.1)	406(18.8)	1635(21.8)	
No partner yet	1639(17.0)	1026(47.6)	613(8.2)	
**Contraceptive use is woman's business**	3305(34.2)	681(31.6)	2625(35.0)	<0.001
**Women contraceptive users become promiscuous**	3536(36.6)	820(38.0)	2717(36.2)	0.039

NDHS: Nigeria Demographic and Health Survey

About 29% of men were contraceptive users while 7500 (77.6%) were non-users. Table 1 further shows the characteristics of contraceptive users and non-users among Nigerian men. The distribution of many background variables varied between the users and non-users. In terms of age distribution, the percentage of users aged 15-29 892 (41.4%) was greater than the percentage of non-users 1242 (16.6%). In terms of education, there was a wide difference between users and non-users. For example, 103 (4.8%) and 1992 (26.6%) of users and non-users respectively have no formal education while 638 (29.6%) and 1275 (17.0%) have higher education. These disparities were also notable for occupation, type of residence, geopolitical region, and fertility desire (p<0.05). When asked about their perception of contraceptives, most of the contraceptive non-users 2625 (35.0) believed contraceptive use is a woman´s business (p<0.001) and that women contraceptive users are promiscuous (p<0.039) (Table 1).

**Perception of non-users: use of contraceptive is women´s business:** we further explored the factors associated with contraceptive perceptions of male non-users (Table 2, Table 3). Age 30-44 years was the peak age group of contraceptive non-users who held the perception that it is women´s business. About one-third of non-users had no formal education (34.1%) while a similar proportion attained a secondary education level (34.5%). Most non-users were involved in agriculture/manual occupation (64.1%) and practiced Islamic religion (62%) (Table 2). The North West (30.9%) and South West (20.7%) had a larger share of the sample. Forty-six percent of non-users had been exposed to family planning messages. Seven out of ten male non-users want more children.

**Table 2:** factors associated with the perception that “contraceptive use is woman’s business” among Nigerian male non-users, NDHS, 2018

Variables	n (%)	Unadjusted OR (95% CI)	Adjusted OR (95% CI)
**Age group in years**			
15-29	446 (17.0)	1.00	1.00
30-44	1398 (53.3)	0.80 (0.70 – 0.92) *	0.99 (0.84 – 1.16)
45-59	780 (29.7)	0.79 (0.68 – 0.92) *	0.98 (0.79 – 1.20)
**Educational level**			
No formal education	896(34.1)	1.00	1.00
Primary	468(17.8)	0.6(0.51 - 0.70) *	0.86(0.73 - 1.02)
Secondary	913(34.5)	0.55(0.48 - 0.63) *	0.79(0.67 - 0.93) *
Higher	348(13.3)	0.4(0.34 - 0.48) *	0.58(0.47 - 0.73) *
**Occupation**			
Not working	50(1.9)	0.98(0.68 - 1.42)	0.71(0.47-1.07)
Professional/services	536(20.4)		
Sales	356(13.6)	1.17(0.97 - 1.39)	0.96(0.80 - 1.16)
Agriculture/manual	1682(64.1)	1.60(1.40 - 1.84) *	1.19(1.02 - 1.39) *
**Religion**			
Catholics	186(7.1)	1.00	1
Other Christians	783(29.8)	1.72(1.40 - 2.11) *	1.22(0.98-1.51)
Islam	1629(62.1)	2.48(2.00 - 3.06) *	1.11(0.86-1.43)
Others	27.5(1.1)	2.53(1.42 - 4.51) *	1.90(1.09-3.30) *
**Wealth index**			
Poorest	600(22.9)	2.42(1.96 - 2.98) *	1.18(0.89-1.55)
Poorer	656(21.5)	1.91(1.57 - 2.33) *	1.10(0.86-1.41)
Middle	487(18.6)	1.33(1.09 - 1.62) *	0.95(0.76-1.18)
Richer	458(17.4)	1.1(0.90 - 1.34)	0.95(0.78-1.17)
Richest	514(19.6)		
**Rural residence**	1605(61.2)	1.66(1.43 - 1.94) *	1.10(0.93-1.30)
**Region**			
North Central	344(13.1)	0.997(0.79 - 1.26)	0.90(0.70-1.15)
North East	527(20.1)	1.63(1.29-2.06) *	1.22(0.93-1.58)
North West	812(30.9)	1.48(1.21 - 1.82) *	1.13(0.87-1.45)
South East	109(4.2)	0.33(0.25 - 0.43) *	0.30(0.22 - 0.42) *
South South	289(11.0)	1.20(0.93 - 1.55)	1.06(0.81-1.38)
South West	543(20.7)	1.00	
**Exposure to family planning messages**	1205(45.9)	0.78(0.69 - 0.88) *	1.04(0.92-1.18)
**Marital status**			
Never married	171(6.5)	1.21(0.99 - 1.47)	1.35(0.98-1.86)
Monogamy	1973(75.2)		
Polygamy	426(16.2)	1.33(1.15 - 1.54) *	0.99(0.81-1.21)
Formerly married	55(2.1)	1.47(1.05 - 2.06) *	1.39(0.97-2.00)
**Number of women fathered with**			
Never fathered	316(12.1)		
One	1680(64.0)	0.81(0.70 - 0.95)	0.93(0.75-1.17)
Two or more	628(24.0)	1.06(0.88 - 1.26)	0.89(0.67-1.19)
**Mobility (past 12 months)**			
Never away	1211(46.2)		
Away < 1 month	792(30.2)	0.90(0.79 - 1.03)	0.85(0.74-0.98)
Away ≥ 1 month	622(23.7)	1.31(1.14 - 1.51) *	1.13(0.98-1.30)
**Fertility preference**			
Wants (more) children	1828(70.0)	0.91(0.76 - 1.08)	1.19(1.02-1.39) *
Undecided	67(2.6)	0.74(0.51 - 1.06)	1.18(0.84-1.65)
Wants no more children	503(19.2)	0.65(0.53 - 0.79)	1
No partner yet	226(8.6)	1	

* Statistically significant; NDHS: Nigeria Demographic and Health Survey

**Table 3:** factors associated with the perception that “contraceptive users are promiscuous” among Nigerian male non-users, NDHS, 2018

Variables	n(%)	Unadjusted OR (95% CI)	Adjusted OR (95% CI)
**Age group in years**			
15-29	504 (18.6)	1.00	1.00
30-44	1438 (52.9)	0.78 (0.69 – 0.90) *	0.97 (0.83 – 1.13)
45-59	775 (28.5)	0.78 (0.67 – 0.90) *	1.00 (0.84 – 1.19)
**Educational level**			
No formal education	871(32.1)		
Primary	497(18.3)	0.79(0.67 - 0.92) *	0.98(0.82-1.17)
Secondary	989(36.4)	0.67(0.59 - 0.77) *	0.84(0.71-0.99) *
Higher	359(13.2)	0.53(0.45 - 0.63) *	0.73(0.59-0.90) *
**Occupation**			
Not working	55(2.4)	1.36(0.99 - 1.89)	1.10(0.77-1.57)
Professional/services	533(19.6)		
Sales	377(13.9)	1.12(0.94 - 1.33)	0.99(0.83-1.18)
Agriculture/manual	1742(64.1)	1.54(1.34 - 1.76) *	1.22(1.05-1.41) *
**Religion**			
Catholics	215(7.9)		
Other Christians	895(33)	1.66(1.37 - 2.02) *	1.52(1.25-1.87) *
Islam	1583(58.3)	1.92(1.59 - 2.33) *	1.35(1.06-1.71) *
Others	23(0.9)	1.83(1.06 - 3.16) *	1.56(0.90-2.69)
**Wealth index**			
Poorest	607(22.3)	2.19(1.81 - 2.64) *	1.56(1.22-2.00) *
Poorer	564(20.8)	1.72(1.44 - 2.06) *	1.36(1.09-1.69) *
Middle	573(21.1)	1.50(1.26 - 1.78) *	1.29(1.05-1.57) *
Richer	492(18.1)	1.24(1.04 - 1.48) *	1.15(0.95-1.38)
Richest	480(17.7)		
**Rural residence**	1615(59.5)	1.3(1.14 - 1.48) *	0.91(0.79 - 1.06)
**Region**			
North Central	363(13.4)	1.02(0.81 - 1.27)	0.96(0.75-1.21)
North East	628(23.1)	2.12(1.70 - 2.65) *	1.60(1.24-2.08) *
North West	721(26.5)	1.10(0.90 - 1.34)	0.86(0.67-1.10)
South East	253(9.3)	0.79(0.63 - 0.98)	0.77(0.61-0.97) *
South South	307(11.3)	1.17(0.93 - 1.47)	1.04(0.82-1.32)
South West	446(16.4)	1.00	
**Exposure to family planning messages**	1257(46.3)	0.92(0.83 - 1.03)	1.16(1.03-1.31) *
**Marital status**			
Never married	216(7.9)	1.41(1.17 - 1.71) *	1.57(1.15-2.15) *
Monogamy	2013(74.1)		
Polygamy	431(15.9)	1.23(1.07 - 1.42) *	1.00(0.82-1.22)
Formerly married	57(2.1)	1.30(0.91 - 1.86)	1.39(0.94-2.05)
**Number of women fathered with**			
Never fathered	360(13.2)		
One	1729(63.6)	0.74(0.64 - 0.86) *	0.90(0.72-1.12)
Two or more	628(23.1)	0.93(0.78 - 1.11)	0.94(0.71-1.26)
**Mobility (past 12 months)**			
Never away	1162(42.8)		
Away < 1 month	919(33.8)	1.06(0.93 - 1.20)	1.00(0.88-1.14)
Away ≥ 1 month	636(23.4)	1.26(1.10 - 1.45) *	1.10(0.96-1.27)
**Fertility preference**			
Wants (more) children	1863(68.6)	0.79(0.66 - 0.94)	1.29(1.11-1.50) *
Undecided	114(4.2)	1.29(0.95 - 1.76)	2.27(1.67-3.08) *
Wants no more children	467(17.2)	0.56(0.46 - 0.68)	1
No partner yet	273(10)		

* Statistically significant; NDHS: Nigeria Demographic and Health Survey

As shown in Table 2, in the bivariate analysis, some independent variables were associated with the perception that the use of contraceptives is women´s business. These include age, education, occupation, religion, wealth index, type of residence, geo-political region, exposure to family planning (FP) messages, marital status, mobility, and fertility preference. The final results from the multivariable model showed that the independent factors associated with the perception that contraceptive is a woman´s business among male non-users include age, education, occupation, religion, geo-political region, and fertility preference. Compared to those aged 15-19 years, older age groups were less likely to hold a negative perception. Further, those who had a secondary (aOR=0.79, CI: 0.67-0.93) and higher education (aOR=0.58, CI: 0.47-0.73) were less likely to perceive that contraceptive use is a woman´s business compared to those who had no formal education. Agricultural/manual workers (aOR=1.19, CI: 1.02-1.39) were more likely to have negative perceptions compared to professionals or those who engaged with the services sector. Similarly, men in other religions (aOR=1.90, CI: 1.09-3.30) were two times as likely as Christians to hold the negative perception. The only regional differential was observed between men from South East (aOR=0.30, CI: 0.22-0.42) and South West. Other regions were not different from the latter. Lastly, men who want more children were more likely to believe that contraceptive use is a woman´s business (aOR=1.19, CI: 1.02-1.39).

**Perception of non-users; women who use contraceptives are promiscuous:** having controlled for other variables, men non-users who had a secondary (aOR=0.84, CI: 0.71-0.99) and higher (aOR=0.73, CI: 0.59-0.90) education levels were less likely than the non-educated to hold the negative perception. Men who were engaged in agricultural and manual work (aOR = 1.22, CI: 1.05 - 1.41) were more likely than professional workers to have the perception that women who use contraception are promiscuous. Furthermore, respondents of the Islamic faith (aOR = 1.35, CI: 1.06 - 1.71) and other Christians (aOR = 1.52 CI: 1.25 - 1.87) were more likely to have a negative perception, of relatives of Catholics. The likelihood of this negative perception declined with the household wealth index. While those in the South-East were less likely to have the perception (aOR = 0.77, CI: 0.67 - 0.97), those in the North-East had a higher likelihood (aOR = 1.60, CI: 1.24 - 2.08) compared to those in the Southwest region. Men who have been exposed to some form of family planning messages had a higher likelihood of having the perception that women contraceptive users are promiscuous (aOR = 1.16, CI: 1.03 - 1.31). Men who wanted more children (aOR=1.29, CI: 1.11-1.50) and the undecided (aOR=2.27, CI: 1.67-3.08) were more likely to have negative perceptions compared to those who wanted more children. This is shown in Table 3.

Two thousand seven hundred and sixteen (36.2%) men among non-users of contraceptives believed that women who use contraceptives are promiscuous. The distribution of background characteristics was similar to that of men who believed that contraceptive is a woman´s business; likewise, the pattern of bivariate association. Independent factors associated with this negative perception were: education, occupation, religion, wealth index, geo-political region, exposure to FP messages, single marital status, and undecided fertility preference.

**Factors associated with non-use of contraceptives among Nigerian men:** Table 4 shows results on factors associated with the non-use of contraceptives among Nigerian men. Univariate models showed that the perception that FP is a woman´s business (OR=1.22, CI: 1.08-1.37) and most of the background variables had bivariate associations with the non-use of contraceptives among Nigerian men. In the final multivariable logistic regression model, age, education, religion, region, exposure to FP messageassociationss, marital status, mobility, and fertility preference show statistically significant association with non-use of contraceptives among Nigerian men. Those who opined that women contraceptive users are promiscuous were less likely to be non-users (OR=0.85, CI: 0.74, 0.96). Young men aged 20-24 and 25-29 years were less likely to be non-users. However, there was an inverse relationship between educational attainment and contraceptive non-use such that the odds of the latter decreased with education. Nigerian men who practiced Islam had a higher likelihood of being non-users of contraceptives (aOR = 1.50, CI: 1.19 - 1.88). Men from the North-East (aOR = 1.39, CI: 1.06 - 1.81), North-West (OR = 1.64, CI: 1.21 - 2.21), and South-East (aOR = 1.44, CI: 1.16 - 1.78) region were more likely to be non-users compared to those from the South West region. Exposure to FP messages was associated with a lesser likelihood of contraceptive non-use (aOR=0.65, CI: 0.58-0.74). Men who want more children had a higher likelihood of being non-users (aOR = 1.66, CI: 1.40 - 1.97) of contraceptives.

**Table 4:** factors associated with non-use of contraceptives among Nigerian men, NDHS, 2018

Variables	Unadjusted OR (95% CI)	Adjusted OR (95% CI)
**Perception**		
Contraceptive use is woman's business	1.22(1.08 - 1.37) *	1.11(0.96-1.27)
Contraceptive users become promiscuous	0.90(0.81 - 1.00)	0.85(0.74-0.96) *
**Age group in years**		
15-29	1.00	1.00
30-44	0.79 (0.71 – 0.88) *	1. 26 (1.06 – 1.48) *
45-59	0.81 (0.72 – 0.92) *	1.66 (1.32 – 2.07) *
**Educational level**		
No formal education	0.29(0.22 - 0.38) *	0.53(0.39 - .70) *
Primary	0.13(0.11 - 0.17) *	0.45(0.35 - 0.59) *
Secondary	0.11(0.08 - 0.13) *	0.39(0.29 - 0.52) *
Higher		
**Occupation**		
Not working	0.41(0.32 - 0.52) *	1.12(0.83 - 1.52)
**Professional/services**		
Sales	1.44(1.21 - 1.72) *	1.00(0.82 - 1.21)
Agriculture/manual	1.73(1.53 - 1.96) *	1.06(0.92 - 1.23)
**Religion**		
Catholics		
Other Christians	0.90(0.77 - 1.05)	0.98(0.82 - 1.17)
Islam	2.75(2.30 - 3.30) *	1.50(1.19 -1.88) *
Others	2.27(1.27 - 4.05) *	1.17(0.63 - 2.20)
**Wealth index**		
Poorest	6.76(5.20 - 8.78) *	1.85(1.31 - 2.59) *
Poorer	2.71(2.26 - 3.26) *	1.27(1.00 - 1.61)
Middle	1.74(1.50 - 2.04) *	1.18(0.98 - 1.43)
Richer	1.19(1.03 - 1.37) *	0.97(0.83 - 1.14)
Richest		
**Rural residence**	1.77(1.55 - 2.03) *	1.15(0.99 -1.35)
**Region**		
North Central	1.15(0.96 - 1.39)	0.77(0.63 - 0.94)
North East	2.88(2.25 - 3.69) *	1.39(1.06 - 1.81) *
North West	4.98(3.75 - 6.60) *	1.64(1.21 - 2.21) *
South East	1.13(0.94 - 1.38)	1.44(1.16 - 1.78) *
South South	0.88(0.73 - 1.06)	1.20(0.98 - 1.49)
South West		
**Exposure to family planning messages**	0.62(0.55 - 0.69) *	0.65(0.58 - 0.74) *
**Marital status**		
Never married	0.10(0.08 - 0.11) *	0.13(0.09 - 0.18)
Monogamy		
Polygamy	1.75(1.40 - 2.19) *	1.03(0.77 - 1.38)
Formerly married	0.54(0.38 - 0.77) *	0.61(0.42 - 0.90) *
**Number of women fathered with**		
Never fathered		
One	5.63(4.98 - 6.38) *	0.72(0.54 - 0.95) *
Two or more	8.37(7.00 - 10.01) *	0.60(0.42 - 0.86) *
**Mobility (past 12 months)**		
Never away		
Away < 1 month	0.82(0.73 - 0.93) *	0.78(0.68 - 0.89) *
Away ≥ 1 month	0.73 - 0.64 - 0.83) *	0.85(0.73 - 0.99) *
**Fertility preference**		
Wants (more) children	11.36(9.86 - 13.08) *	1.66(1.40 - 1.97) *
Undecided	7.90(5.62 - 11.09) *	1.32(0.93 - 1.86)
Wants no more children	5.99(5.15 - 6.96) *	1
No partner yet		

* Statistically significant; NDHS: Nigeria Demographic and Health Survey

## Discussion

Previous studies on contraceptive use in Nigeria have often focused on women. This study provides information on male contraceptive non-users. This study documented the prevailing negative perceptions about contraceptive use among Nigerian men. Our study showed that almost three-quarters of the men were not using contraceptives. A multi-country study on exposure to family planning messages and modern contraceptive use among men in urban Kenya, Nigeria, and Senegal also reported a high prevalence of contraceptive non-users [24]. This persistent high prevalence could be attributed to the low level of exposure to family planning messages among men [19].

One-third of contraceptive non-users among the men perceived that contraceptive is a woman´s business. This finding corroborates the outcome of Kabagenyi *et al*. and Jangu in Uganda where the male participants reported that family planning, issues surrounding children, and childbirth are strictly meant for women, not men [15,16]. Other studies conducted in rural Southern and Northern parts of Nigeria are also in agreement with our findings [25,26]. Low level of education; lack of awareness, incorrect knowledge, and information about contraceptives are possible reasons for this wrong perception among Nigerian men [24,26].

Our study further showed that a high proportion of contraceptive non-users reported that contraceptives make women promiscuous. A qualitative study in Uganda documented a similar report where some men stated that contraceptive usage may encourage extramarital affairs among women [16]. Studies especially from Northern Nigeria also documented men´s negative perception towards the use of contraceptives [16,25,26] and found that women are likely to be immoral and have multiple sexual partners while using contraceptives. This is possible because men do not consider contraceptive use acceptable for faithful married women; they believe it increases the woman´s potential for infidelity. Hence, they project a stigmatizing belief that contraceptive was most often used in the contexts of the female commercial sex trade [16,17,19,27,28]. On the contrary, in a qualitative study by Dral *et al*. in Malawi, the men stated that their women cannot be promiscuous because the Malawian law emphasizes that women cannot have multiple partners, only men can [29]. However, most other studies reported men´s averseness to contraceptive use by women [15,30].

Our study showed that lower education, farmers/manual workers, Islamic faith, and residents in the North East of Nigeria, never heard about family planning messages and wanting more children predisposed men to hold the perception that contraceptive makes women promiscuous. Studies have shown that higher education increases approval and acceptability of family planning among men, that is if men are well aware of family planning, they will be more supportive and involved [31,32]. Islamic religion has been reported to negatively influence contraceptive use, especially in the Northern part of Nigeria [26,30,33,34]. The religious convictions of the Islamic faithful are that God´s injunction for human beings is to reproduce of their kind and populate the earth [26,30]. Other previous studies among men in Nigeria reported that men´s desire for many children is seen as a form of economic support; to extend the family lineage and at the same time to boost the social status of the household head [33,35,36]. These factors constitute a huge barrier to contraceptive uptake and probably account for low CPR in Nigeria [9,37,38].

Our study also found that men who were non-users of contraceptives were older, had lower education, were of Islamic faith, needed family planning messages, wanted more children, and were residents in the Northeast and Western regions of the country. This showed a regional variability of non-use of contraceptives among men in the Northern part of Nigeria as compared with other parts of the country. This variation can be attributed to low education, religion, cultural beliefs, and socioeconomic status [10,30,39].

**Limitations of the study:** the cross-sectional nature of this study makes it difficult to establish causality. Some important variables that could have been used to comprehensively measure perception about contraceptives were not included in the original data, thus primary data may be necessary to address these issues. Furthermore, the objectives of this study can better be explored with a longitudinal study, this is therefore suggested for future research. However, this study has been able to highlight the huge presence of male contraceptive non-users in Nigeria, their perceptions, and characteristics.

## Conclusion

This study emphasized the prevailing negative perceptions about FP among Nigerian men which strongly impact family planning uptake. These were attributed to low education, older age, religion, manual workers, desire for many children, and region of residence. It is therefore essential to create and improve awareness and knowledge about contraceptive use among men. Stakeholders of religious bodies should be engaged in strategies that will deploy contraceptive messages to men in worship places and at the regional level. It is essential for policymakers and program implementers to develop educational interventions to address negative perceptions and consequently increase contraceptive uptake.

### What is known about this topic

- *Knowledge and uptake of contraceptives;*
- *Prevalence of contraceptive use among women/men;*
- *Types of contraceptives people uptake*.

### What this study adds

- *Characteristics of contraceptive non-users;*
- *Significant predictors of non-use of contraceptive;*
- *Non-users’ perception about contraception and family planning*.
